# Wide-field and non-invasive imaging of brain tumours with scattered light techniques

**DOI:** 10.1364/BOE.587407

**Published:** 2026-02-02

**Authors:** Philip Binner, Jack Radford, Ilya Starshynov, Mansa Madhusudan, Karen Strathdee, Katrina Stevenson, Matthew Walker, Giuseppe Ciccone, Gonzalo Tejeda, Andrew B. Tobin, Massimo Vassalli, Anthony J. Chalmers, Jinendra Ekanayake, Daniele Faccio

**Affiliations:** 1 Advanced Research Centre, School of Physics and Astronomy, University of Glasgow, United Kingdom; 2 Wolfson Wohl Cancer Research Centre, School of Cancer Sciences, University of Glasgow, United Kingdom; 3 Advanced Research Centre, James Watt School of Engineering, University of Glasgow, United Kingdom; 4 Institute for Bioengineering of Catalonia (IBEC), The Barcelona Institute for Science and Technology (BIST), Barcelona, Spain; 5 Advanced Research Centre, School of Molecular Biosciences, University of Glasgow, United Kingdom; 6 Stanford Neuroscience Health Center, Stanford University, USA

## Abstract

The ability to identify tumour tissue in a label-free, contactless, and real-time manner is much needed in tumour resection surgery. Current techniques cause interruptions to surgical flow and have high false positive rates, which can cause collateral damage to healthy brain tissue. We propose laser light scattering techniques, such as diffuse correlation spectroscopy and laser speckle contrast imaging, to image mechanical stiffness differences in the brain’s surface associated with tumour tissue. We validate the optimal processing technique quantitatively with a controlled experiment in which paraformaldehyde was used to induce a change in tissue stiffness in ex vivo mouse brains. We then demonstrate that the technique applies to tumour localisation using ex vivo mouse models with real tumours. Qualitative comparisons with magnetic resonance imaging indicate accurate tumour localisation using only surface stiffness changes to underlying tumours. We also demonstrate sub-millimetre precision when imaging brain slices.

## 1. Introduction

Intraoperative imaging plays an important role in the surgeon’s ability to fully resect a tumour whilst limiting collateral damage to healthy tissue. However, the only clinically approved tools for the imaging of tumours in the operating room currently are intraoperative magnetic resonance imaging (MRI) and fluorescence imaging with 5-aminolevulinic acid. These current methods have some drawbacks. For example, intraoperative MRI interrupts the surgeon’s workflow and increases operation time [1–3]. On the other hand, fluorescence-guided surgery with 5-aminolevulinic acid is not effective for all tumour sub-types, such as low-grade gliomas and pituitary tumours, and is reported to have high false positive rates in non-tumourous tissues [4,5]. There is, therefore, a need for alternative tools that aid surgeons in interventions. Optical tools, besides fluorescence-guided surgery, are emerging for intraoperative tumour imaging due to their versatility and low cost. Emerging techniques such as hyperspectral imaging [6,7], diffuse reflection spectroscopy [8–11], structured light imaging [12,13], optical coherence tomography [14], fluorescence lifetime imaging [15–17], and Raman spectroscopy [18,19] for image-guided surgery map tumourous tissue based on their optical properties or blood oxygenation [20,21].

Changes in tissue stiffness are correlated with cancer progression [22–24] and are often used by clinicians as a proxy for tumour tissue during operations. Indeed, magnetic resonance elastography (MRE), an extension of MRI that is sensitive to tissue stiffness, has been used to diagnose brain tumours and aid presurgical planning [23,25,26]. Devices that can assess brain stiffness present an intuitive and valuable technology for surgical use. Laser speckle interferometry has been used extensively in rheology [27–36] and we have previously shown that diffuse correlation spectroscopy (DCS) can infer brain tissue stiffness [37]. However, the principle of using DCS and related approaches to assess stiffness has not yet been validated in brain tumour tissue.

DCS is a computational sensing and imaging method that uses scattered light to probe dynamics, such as blood flow or tissue Brownian motion [38–42]. The technique analyses the temporal autocorrelation of the speckle pattern generated by multiple light scattering of long coherence length laser light from a sample. Here, the main DCS measurement parameter, the speckle decorrelation time $\tau_{c}$ , may be used to indirectly measure tissue stiffness [43]. Laser speckle contrast imaging (LSCI) is a light scattering technique similar to DCS. However, instead of autocorrelation, LSCI applies a temporal moving window across a speckle pattern time series and computes the speckle contrast K . Like $\tau_{c}$ in DCS, the speckle contrast may also be used to infer tissue stiffness, with some experiments utilising ultrasound to drive vibrations in the sample [44,45].

Here, we extend the optical assessment of brain tissue stiffness to preliminary applications in imaging tumour tissue. We perform a proof-of-concept study demonstrating that light scattering techniques such as DCS and LSCI may be used to image tumour from healthy brain tissue in mouse models over a wide field of view.

For measurements on samples with slow dynamics, DCS requires longer measurement times to correctly estimate $\tau_{c}$ . Empirical estimates show that one needs to acquire for a period $∼100\tau_{c}$ [37]. In this work, we make improvements to DCS for imaging applications through the use of a machine-learned approach, machine learning diffuse correlation spectroscopy (ML-DCS) based on a convolutional neural network (CNN), which can accurately produce a map of $\tau_{c}$ across the brain given only short acquisition times of <10 s, i.e., only $∼10\tau_{c}$ . We also show that this method can determine tumour localisation with improved signal-to-noise ratio (SNR) compared to conventional DCS post-processing. The ML-DCS and LSCI approaches show comparable ability to identify tumour tissue in both tumour proxy and tumour model experiments. These results indicate that light-based rheometer techniques are viable candidates for tumour imaging and support the need for future work to move towards human tissue experiments.

## 2. Methods

### 2.1. Mouse brain sample preparation and imaging

Two mouse models were used in this work. In preliminary proof-of-concept experiments to validate our approach, we used wildtype mouse brain samples provided by the Centre for Translational Pharmacology at the University of Glasgow. Mice were sacrificed by cervical dislocation followed by extraction of the brain. The extracted brains were then injected with a 0.02 ml solution of 4% paraformaldehyde (PFA) (Thermo Fisher Scientific) and fluorescent dye 4’,6-diamidino-2-phenylindole (DAPI) (Thermo Fisher Scientific). Here, the PFA locally fixes a region of the brain, serving as a tumour proxy, and DAPI, when illuminated with UV light, provides a reference indicating areas of increased stiffness. The PFA is mixed with DAPI according to the recipe in [46]. White light, ultraviolet (UV) excitation, and laser speckle acquisitions of the top view of the brain samples were obtained at an exposure time of 9500 µs and a frame rate of 100 fps for a total acquisition time of 60 s. Each acquisition for the different light sources mentioned above was taken sequentially. The PFA-DAPI mixture is optically transparent and was chosen so that there is no visible difference in the brain before and after injection, either to the naked eye or through white light imaging.

Nanoindentation measurements on the PFA-injected mouse brains were also performed to provide a mechanical ground truth of tissue stiffness. Young’s modulus measurements were obtained using a mechanical nanoindenter (Optics11Life Chiaro) for two fixed brain slices and seven fresh brain slices. The brains were sliced into 1 mm thick samples before measurements with the nanoindenter. All nanoindentations were performed on the hippocampus, a large and easily identifiable region of the brain, on which we could perform measurements consistently. Effective Young’s modulus values were obtained using Optics11Life’s DataViewer software [37].

For further validation of the optical techniques on real tumour samples, 005/Bl6 mouse model samples [47,48] were provided by the Wolfson Wohl Cancer Research Centre at the University of Glasgow. One day prior to mouse culling, whole brain MRI scans were acquired (1 Tesla, T2 Fast Spin Echo 3D Axial Sequences, 1 mm slice thickness, 2000 ms repetition time, 83.7 ms echo time, $90^{o}$ flip angle). Mice were maintained under inhaled isoflurane anaesthesia (induction 5% v/v; maintenance 1.5–2.0% v/v) in the medical air during the imaging procedure (duration ∼ 30 minutes). On the day of optical measurements, the mice were culled and transported to the Advanced Research Centre, University of Glasgow for imaging.

Optical imaging of the 005/Bl6 model was performed in two steps. First, a top view acquisition of the entire brain was obtained following the white light, UV, and fluorescent imaging protocol outlined above. Then, for comparison with MRI slices and further inspection, the brains were sectioned into 1 mm slices, and their cross-sections were imaged. Imaging the brain cross-sections was necessary, as tumours were around 1-2 mm deep in the tissue.

For preparation of the tumour loaded 005/Bl6 model, 005 cells were routinely cultured as spheres in Dulbecco’s Modified Eagle’s Medium F12 supplemented with 2 mM glutamine, 20 ng/mL EGF, 20 ng/mL FGF, 1% N2 supplement, 1% penicillin/streptomycin (all from Thermo Fisher Scientific), and 4 µg/ml heparin (Sigma). All cell cultures were maintained at $37^{o}$ C, 5% ${\mathrm{CO}}_{2}$ routinely passaged every 3-4 days. They were also routinely tested for mycoplasma. Female C57Bl/6 mice were orthotopically injected with 005 cells suspended in phosphate buffered saline solution into the subventricular zone as previously described [49]. Mice were maintained under inhaled isoflurane anaesthesia (induction 5% v/v; maintenance 1.5–2.0% v/v) in the medical air during the surgical period ( ∼ 20 minutes). Mice were monitored for the duration of the experiment and humanely sacrificed when they showed neurological (hemiparesis, paraplegia) or general symptoms (hunched posture, reduced mobility, and/or weight loss >20%).

All animal experiments were performed under the UK Home Office Project Licence and carried out with ethical approval from the University of Glasgow under the Animal (Scientific Procedures) Act 1986 and the EU directive 2010. Mice were maintained in individually ventilated cages with environmental enrichment, and ARRIVE guidelines were followed.

### 2.2. Experimental apparatus and optical data acquisition

**Fig. 1.**
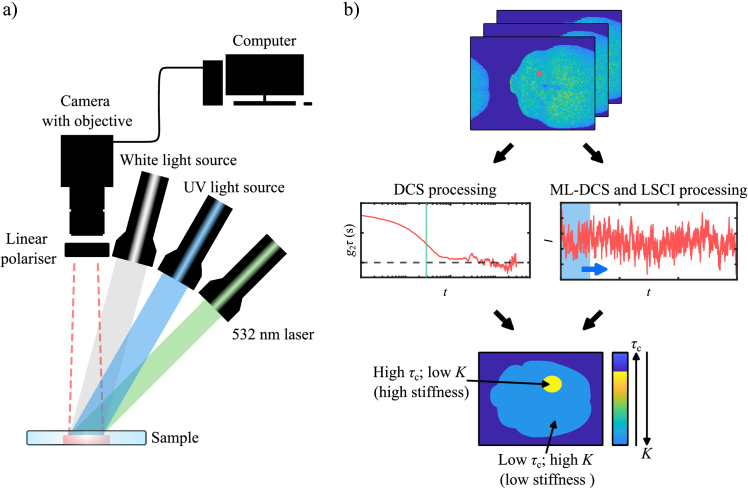
**Experimental Setup and Data Processing Pipeline.** a) A diagram of the imaging apparatus. Illumination is performed sequentially using a white light source for a white light image, a UV light source for a fluorescence image, and a laser source for the DCS, ML-DCS and LSCI images. The illumination sources were positioned at an oblique angle to the sample. A CMOS camera imaged the light reflected and scattered off the sample, and a computer performed computational imaging with this light. Light from the sample was cross-polarised (10,000:1 extinction ratio) to reduce specular reflections from the sample. Here, linearly polarised light illuminated the sample, and a linear polariser was mounted on the camera objective and turned until specular reflections were eliminated from the brain’s image before speckle acquisition. b) A speckle series of the brain obtained by laser light illumination is shown with a single pixel highlighted in red. For DCS processing, the $\tau_{c}$ of the sample pixel is found by means of $g_{2}$ time series autocorrelation. For LSCI, a window is scanned along the time series and K is found, respectively. The window size is different for both techniques. The above processes are performed for each pixel, and the output image is a colourmap of either $\tau_{c}$ or K .

A diagram of the experimental setup is shown in Fig. 1(a). For measurements on the mouse tumour proxy model, three light sources consisting of a white light, a 350 nm collimated UV light, and a 532 nm continuous wave (CW) laser source were used. Light scattered from the sample was cross-polarised to limit specular reflections and was collected with an sCMOS camera (Basler acA2440-75uc for the tumour proxy model and Basler acA1920-155um for the 005/BL6 tumour model). Different light sources illuminated the sample sequentially. For the CW 532 nm source, a 60 s acquisition of the brain’s reflected laser speckle was obtained at 100 fps and at an exposure of 9500 µs. Speckle series data was then processed by a computer for DCS, ML-DCS, and LSCI analyses to finally obtain a colour map of either speckle decorrelation time $\tau_{c}$ for DCS-type analyses or temporal speckle contrast K for LSCI.

The 005/Bl6 mouse tumour model was processed in a similar manner as the PFA-injected mouse brain model. However, the UV light source was not necessary, as the PFA-DAPI mixture was not used. Additionally, cross-sections of the mouse brains were obtained for comparison of optical and MRI measurements. Cross-sections were 1 mm slices cut with a vibratome (Leica VT 1200S).

### 2.3. DCS, ML-DCS, and LSCI data processing

Figure 1(b) illustrates the data processing pipeline. A speckle time series of the mouse brain was post-processed using DCS, ML-DCS, and LSCI. For DCS processing, the $g_{2}$ autocorrelation was applied pixel-wise to each pixel time series. The $g_{2}$ function is defined as,

(1) $$g_{2}(\tau )=\frac{\left\langle I(t)I(t+\tau )\right\rangle }{\langle I(t)\rangle^{2}}.$$

I(t) and I(t+τ) represent a time series with no time lag and a time lag of τ , respectively. Angle brackets represent a time average. The output of the $g_{2}$ function is a decorrelation curve, from which $\tau_{c}$ may be obtained and used to infer tissue stiffness. A $\tau_{c}$ value for each pixel is found by taking the 1/ e value relative to $g_{2}(0)$ for each $g_{2}$ curve, using

(2) $$\tau_{c}=\underset{\tau }{\mathrm{argmin}}\left|g_{2}(\tau )-\left[1+\frac{\left(g_{2}(0)-1\right)}{e}\right]\right|.$$

We note here that $\tau_{c}$ is independent of the $g_{2}(0)$ , however, in rare cases where pixels have very low dynamic range i.e., low $g_{2}(0)$ , we would expect more uncertainty in the $\tau_{c}$ estimate for that pixel.

For the ML-DCS method, a CNN akin to that used in [50] was trained on 100 fps, 10 s synthetic noiseless Brownian motion time series data and applied to 10 s temporal windows of the speckle series data. Since the total acquisition time is 60 s and the ML-DCS model determines $\tau_{c}$ from 10 s windows, multiple ML-DCS outputs were averaged to improve the SNR of the output images. The architecture and training of the ML-DCS model are described in more detail in the Supplement 1. However, to summarise the model, it maps a single 10 s time series input to an output $\tau_{c}$ . The model was trained on a uniform distribution of $\tau_{c}$ from 0 to 2 s, as we have empirically found that mouse brain surface $\tau_{c}$ measurements typically sit within this range. The model was trained with a 90:10 training-validation split until its mean absolute percentage error fell below 5% with no signs of overfitting. Since the model was trained on synthetic data, real brain time series data were first denoised using a Butterworth filter of order 1 and a critical frequency of 0.1 before being fed into the ML-DCS model for inference of the brain’s $\tau_{c}$ . The inference time for a single frame was ∼2 s (RTX 4070) for a 10 s time series. The ML-DCS model was trained on synthetic samples undergoing Brownian motion. The model itself cannot generalise to non-Brownian motion data, however, we show in the coming sections that the technique is able to differentiate between soft and stiff tissue. A future improvement to the model could, therefore, be in training it with non-Brownian and viscoelastic effects.

For LSCI, K for a temporal scanning window of width seven frames was calculated using,

(3) $$K=\frac{\sigma }{\left\langle I\right\rangle },$$

where σ is the standard deviation of the single pixel time series window and ⟨I⟩ is the mean. This calculation was performed pixel-wise across the whole speckle series and the mean for all windows across time was used for the final LSCI image. The seven frame window is a trade-off between computational speed and sensitivity to the tumour region of interest (ROI) and was chosen by studying output for temporal window lengths of 3 to 51 frames. A smaller temporal window generally outputs a less accurate speckle contrast; a longer window tends to be more accurate but is also susceptible to external noise, such as sample motion. A real example of this could be in a future in vivo measurement, where blood flow, pulsation, and brain movement are in effect. Overall, the temporal window should not be larger than $\tau_{c}$ , as the time series signal would decorrelate over this time.

## 3. Validation on PFA-injected mouse brain

**Fig. 2.**
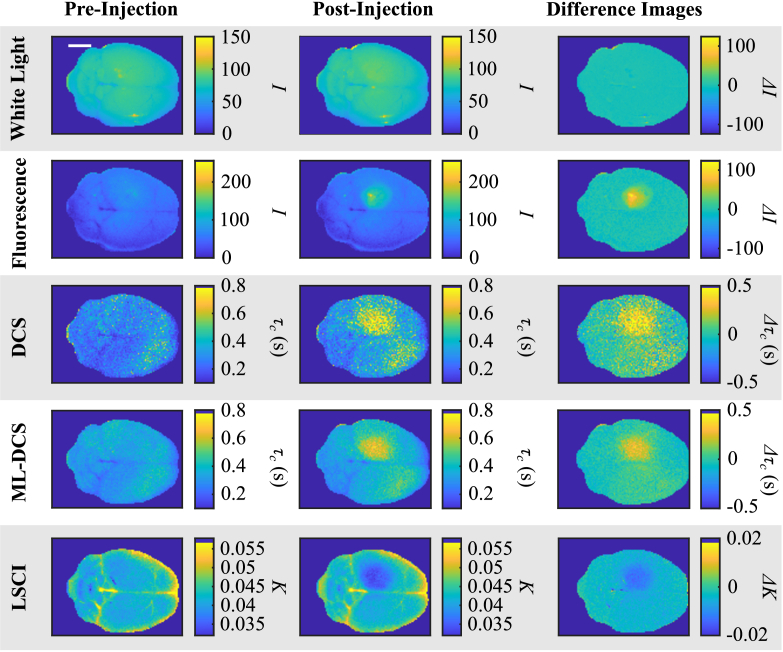
**Tumour Proxy Model DCS, ML-DCS, and LSCI Results.** Row headings indicate imaging methods of white light, fluorescence, DCS, ML-DCS, and LSCI. Column headings indicate if images were taken pre-injection and post-injection of the PFA-DAPI tumour proxy. The last column shows a difference image of the post- and pre-injection images. The scale bar on the top left panel is equivalent to 5 mm and applies to all other panels.

Prior to measurements on real tumour brains, we first explored the efficacy of our optical techniques with a PFA-injected mouse brain, which exhibited a local region of increased stiffness relative to the surrounding healthy tissue. Results for the PFA-injected mouse brain are shown in Fig. 2. The figure contains panels for white light, fluorescence, DCS, ML-DCS, and LSCI before and after the injection of the PFA-DAPI mixture. The last column in the figure panel shows ‘difference’ images obtained by subtracting the pre-injection image from the post-injection image for the five imaging formats mentioned above, and shows a map of changes made to the brain by the PFA-DAPI solution. Each image format here was co-registered. Any misalignment may be seen in the difference images, where values would be offset from zero except for the tumour ROI.

The white light image of the brain shows no changes before and after the injection of the solution, as would be observed by the naked eye or standard intensity imaging formats. The fluorescence image shows, in the post-injection image, a local area where the PFA-DAPI solution has settled on the brain, as indicated by the fluorescence of the DAPI in the solution. This post-injection image therefore, acts as a ground truth for the tumour proxy. We note that in the pre-injection image, there is a weak reflection in the upper brain hemisphere that is due to the oblique illumination of the UV light source.

The DCS and ML-DCS images both show a distinct and well isolated region of high $\tau_{c}$ in the upper hemisphere where the PFA-DAPI solution was injected. The higher $\tau_{c}$ relative to the surrounding healthy tissue indicates that it is stiffer, as expected by the tissue stiffening properties of the PFA in the solution. The ML-DCS has noticeably better SNR by visual inspection. We have also observed that a shorter 1 s acquisition is enough for the ML-DCS model to differentiate stiffened tissue from fresh tissue, however, this is an empirical observation from this dataset and may vary with $\tau_{c}$ , illumination, and camera noise.

The LSCI, on the other hand, reveals a region of low speckle contrast that coincides with the locally stiffened area of the brain. The reason for this is that the exposure time of the camera was set to 9500 µs and is considerably smaller than $\tau_{c}$ , i.e., $T_{\exp}<<\tau_{c}$ . In this case, Eq. (3) computes low contrast for a more static time series (equivalent to stiffer tissue) and high contrast for a fast-changing time series (equivalent to softer tissue). Lastly, the difference images, apart from the white light images, all show that the only difference in the brain was that induced by the injection of the PFA-DAPI stiffening agent.

## 4. Comparison of imaging methods on PFA-injected mouse brains

**Fig. 3.**
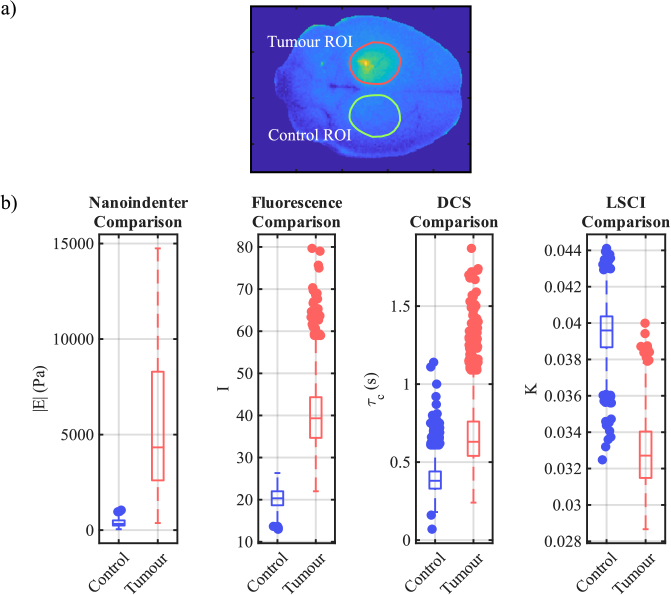
**Statistical Comparison of Methods.** a) The fluorescent post-PFA-injection reference image with tumour and control ROIs annotated. b) Box plots comparing healthy and PFA-injected mouse brain for nanoindentation, fluorescence, DCS, ML-DCS, and LSCI measurements. For each PFA and control comparison, Welch’s t-test p-values were less than 0.0001 in all cases.

We quantified the sensitivity of each imaging method by analysing image statistics in the stiffened and fresh brain regions. A ROI was drawn around the tumour region in the post-PFA-injection fluorescence reference image. This tumour ROI was then mirrored to obtain a corresponding, symmetrically located control ROI in the unfixed (lower) hemisphere. These ROIs are shown in Fig. 3(a). From this data, the SNR of the tumour region was calculated as ${\mathrm{SNR}}_{T}=\mu_{T}/\sigma_{T}$ , and the t -statistic, which measures the separation between the ROI means, was computed as

(4) $$t=\frac{\mu_{T}-\mu_{C}}{\sqrt{\frac{\sigma_{T}^{2}}{n}+\frac{\sigma_{C}^{2}}{m}}}.$$

Here, $\mu_{T,C}$ and $\sigma_{T,C}$ denote the mean and standard deviation of the tumour and control populations, respectively. n and m are their sample sizes, which correspond to individual nanoindentations for the mechanical measurements and to individual pixel values of intensity, $\tau_{c}$ , or K for the optical measurements. This formulation corresponds to Welch’s t -test when n≠m and was implemented using MATLAB’s ttest2 function.

Figure 3(b) shows box plots of control (fresh tissue) versus tumour (fixed tissue) brain regions for different measurement techniques. Here, we have the previous fluorescence, DCS, ML-DCS, and LSCI techniques, as well as Young’s modulus measurements. A tumour ROI is defined by drawing a polygon around the high-fluorescence tumour area in the reference fluorescence PFA-injected mouse brain image. The control ROI is made by mirroring the tumour ROI in the vertical direction to obtain an identical ROI in the control hemisphere of the brain.

Figure 3 presents box plot comparisons of the nanoindenter, fluorescence, DCS, ML-DCS, and LSCI measurements, which show clear differences between the control and PFA-injected mouse brain measurements. Furthermore, p-values less than 0.0001 were obtained via Welch’s t-tests for each of the modalities. Table 1 lists the mean μ and standard deviation σ for the control and tumour proxy measurements, from which $\mathrm{SNR}=\mu_{T}/\sigma_{T}$ of each of the modalities could be obtained. Here, we define SNR as $\mu_{T}/\sigma_{T}$ as opposed $(\mu_{T}-\mu_{C})/\sigma_{T}$ , as in a future clinical setting, one would typically only have access to one image. Moreover, we do not expect to see a difference in trend between the SNRs of each imaging format. We see here that the LSCI has the best SNR for identifying the stiffened tumour region, outperforming the fluorescence and nanoindentation methods. We further note that the SNR of the DCS method could be improved by increasing the acquisition time, although this impacts its medical relevance, as more real-time techniques would be favoured in a medical setting.

**Table 1.** **Comparison of Statistics for Each Technique on PFA-Injected Brains.** Population means, standard deviations, and SNR and t -statistic are compared for the different measurement methods. $\mu_{C,T}$ are mean measurements of the control and tumour populations respectively. Likewise, $\sigma_{C,T}$ are the standard deviation of the control and the tumour populations. ${\mathrm{SNR}}_{T}$ is found by ${\mathrm{SNR}}_{T}=\frac{\mu_{T}}{\sigma_{T}}$ . t is the Welch’s test statistic between the $\mu_{C}$ and $\mu_{T}$ calculated using Eqn. (4).

	$\mu_{C}$	$\sigma_{C}$	$\mu_{T}$	$\sigma_{T}$	${\mathrm{SNR}}_{T}$	t
Nanoindenter	390.76 Pa	206.861 Pa	5369.93 Pa	3801.434 Pa	1.4	12.26
Fluorescence	20.26	2.535	40.14	7.469	5.4	112.18
DCS	0.39 s	0.099 s	0.67 s	0.206 s	3.3	54.28
ML-DCS	0.42 s	0.045 s	0.60 s	0.059 s	10.2	108.35
LSCI	0.04	0.001	0.03	0.002	15.0	126.84

## 5. Application to mouse tumour models

After confirming quantitatively that the three light scattering techniques worked on a controlled experiment using the PFA-injected mouse brain samples, we applied the techniques to a real tumour model. Real tumours tend to be heterogeneous, unlike our earlier PFA-mouse model, and were used to assess the ability of our optical techniques in a more clinically relevant setting. For these samples, MRI scans were performed prior to optical measurements and acted as ground truth references for any optical measurements. For this, LSCI images were qualitatively compared with corresponding MRI images to confirm that our optical approach could accurately determine the tumour regions.

**Fig. 4.**
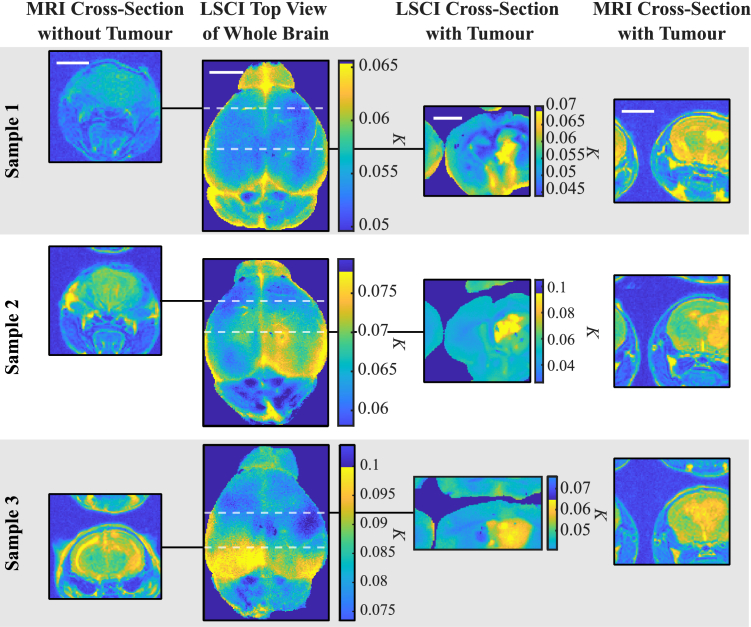
**Tumour Model LSCI and MRI Results.** Whole brain LSCI images are shown for three mouse brain samples. Corresponding cross-sectional images for LSCI and MRI are also shown, which show slices of the brain with (right hand side images) and without (left hand side images) a tumour. Tumours appear as a hotspot in the LSCI maps indicating they are softer than the healthy tissue. Sample 3 shows signs of hydrocephalus through MRI in the brain’s left hemisphere, which is also detected as a high contrast, low stiffness hot spot in the LSCI top view image. White scale bars are equivalent to 5 mm. Note that colourbar ranges are different for each panel.

Optical and MRI results for the 005/Bl6 tumour model are shown in Fig. 4. For the optical images here, we only show LSCI images, as these had the best SNR and t -statistic in the previous analysis. However, ML-DCS images are shown in the Supplement 1 in Fig. S3.

Top view LSCI images are shown for three mouse brain samples. MRI cross-sections without the tumour are shown on the left of the top view images. On the right are LSCI and MRI cross-sections of the brain with the tumour. Horizontal dashed lines indicate where the cross-sectional MRI and LSCI images were obtained.

In each of the three top view LSCI images, the tumour, located in the right hemisphere, shows increased speckle contrast K in the tumour region, corresponding to softer tissue. There is no clearly discernible structure in the K -map in sample 1, whereas in sample 2 there is a clear region of high contrast K that is correlated with tumour position seen in the MRI image. Comparing the MRI cross-sections, the tumour in sample 1 is smaller in volume and deeper in the brain than in sample 2, which accounts for the weak contrast changes on the surface of the brain. Sample 2 has the greatest contrast signal with a maximum K in the right hemisphere, but also high K in the left hemisphere. Referring now to the MRI cross-section, the tumour has a large volume and indeed exists in both hemispheres of the brain but lies predominantly in the right hemisphere. The LSCI and MRI images are therefore in agreement, indicating that the tumour can still be detected at ∼2 mm depth from the outer surface.

For sample 3, there is a large area of increased contrast in the left hemisphere. Upon examining the healthy MRI reference, we determined that this hemisphere suffered from hydrocephalus, an accumulation of fluid in the brain’s ventricles, as shown by the yellow arc-shaped streak. This would account for the very high K measured by LSCI relative to otherwise healthy tissue, as volumes with more fluid would yield softer tissue and smaller values of Young’s modulus. This constitutes a potential false positive in the optical imaging modality, which is also often seen in the gold-standard MRI. In its current state, the technique requires more sensitivity to be used for diagnostic purposes and should be complemented with current diagnostic tools. Lastly, the tumour is not clearly visible in sample 3, probably for similar reasons as in sample 1, i.e., as a result of the slightly larger depth position.

Interestingly, the increased K of the tumour in sample 2 indicates that its tumour tissue was softer than the healthy tissue. To ensure that the top view high K signals represented tumour tissue, we sectioned the brains into 1 mm axial slices and obtained cross-section LSCI images for slices with the high K signal. This is shown in the third column of Fig. 4, where a dashed white line indicates the position of the LSCI section. In all cases, the tumours are now clearly visible, therefore, supporting the hypothesis that the low visibility in samples 1 and 3 is due to the depth of the tumours. One can also ascertain sub-millimetre inhomogeneities in each of the tumours’ structure. Lastly, the tumours in all three samples had increased K (lower stiffness) compared to the healthy tissue. Whilst the common conception is that brain tumours tend to be stiffer than healthy tissue, in reality, tumours have been reported as either stiffer or softer than healthy brain matter [23,24,51–55]. Furthermore, mouse model tumours are known not to fully reproduce all features of human tumours, which could also explain the lower stiffnesses we obtained. In any case, these results indicate that optical stiffness measurements can image changes in tissue stiffness up to depths of ∼1−2 mm below healthy brain tissue.

## 6. Discussion

The results presented here are a preliminary step towards an intraoperative tumour imaging device, leveraging DCS and LSCI to distinguish tumours from healthy tissue based on their different mechanical properties. Future work on this subject should be devoted to optimising the technique in humans and clinical environments. Such future experiments would be in vivo, whereby a device is used in an operating room to image a healthy brain and tumour tissue. Additionally, ex vivo experiments can be performed, but now on excised human tumour tissue.

Another interesting avenue to explore with this method is tumour stiffness-related biomarkers. Although tissue stiffness is a widely used proxy for surgeons to differentiate tumour from healthy tissue, biomarkers related to tissue stiffness are currently not well understood. In vivo and ex vivo measurements would be ideal for such a study, as one could perform relative tissue stiffness measurements using the aforementioned optical techniques and also conduct tissue biopsies to determine, for example, specific proteins present in stiff tumour tissue.

Sensitivity to subsurface tumours would be a valuable improvement to the methods discussed. For example, DCS and LSCI are well suited for time-domain and multispectral analyses. time-domain diffuse correlation spectroscopy (TD-DCS) makes use of cameras capable of time-correlated single photon counting to time tag photons and estimate the depth of light penetration in a sample. This would require more complicated setups, expensive equipment capable of time-tagging photons, and high-sensitivity detectors to overcome the low-photon budget for deeper areas of the brain. Multispectral imaging approaches utilise different laser wavelengths and the brain’s optical transparency to peer deeper into the sample [56]. For example, one could use green light to probe the surface features of the brain, and red and near infrared light to peer deeper into the brain. Although cheaper, one must carefully choose filters and detectors for various wavelengths of light. Moreover, light penetration depth is not linearly dependent on wavelength for biological matter, so more investigation is needed to optimise specific wavelengths to use for brain applications. Both methods may be used to gain additional sensitivity in imaging deep tumours or to construct a volumetric image of a tumour and healthy tissue.

In other respects, the computational algorithms of LSCI could be further improved. For instance, by incorporating non-Newtonian models of the tissue, one could obtain a more accurate differentiation between diseased and healthy tissue by studying their viscoelastic properties [57]. Moreover, time-frequency analysis is also an interesting route and has already been shown to improve the diagnostic capability of LSCI in blood flow applications [58].

## 7. Conclusion

In this work, we demonstrated the applicability of non-labelled and contactless coherent optical techniques to localise tissue stiffness changes and infer tumour position in a mouse brain. We compared DCS and LSCI with a controlled experiment using PFA injections to induce a localised stiffness change in ex vivo mouse brain samples. The high signal-to-noise ratio showed that LSCI had the highest sensitivity to changes in tissue stiffness.

We validated the sensitivity of LSCI to the problem of tumour localisation by testing the technique with three independent ex vivo mouse brains with real tumours. Comparisons with MRI images indicate that LSCI correctly localised stiffness on the brain surface associated with the position of underlying brain tumours. Furthermore, when cross-sectioned, details of tumour heterogeneity could be resolved with sub-millimetre precision.

We envision future developments of this technique to assess tumour localisation with in vivo experiments, and with depth resolution, towards realising a medical device that can demarcate tumour tissue from healthy tissue intraoperatively.

## Supplemental information

Supplement 1 Supplemental Document 1 https://doi.org/10.6084/m9.figshare.31132267

## Data Availability

Data underlying the results presented in this work are available at [59].

## Supplemental document

See Supplement 1 for supporting content.
