# Wide-field and non-invasive imaging of brain tumours with scattered light techniques: erratum

**DOI:** 10.1364/BOE.592667

**Published:** 2026-03-20

**Authors:** Philip Binner, Jack Radford, Ilya Starshynov, Mansa Madhusudan, Karen Strathdee, Katrina Stevenson, Matthew Walker, Giuseppe Ciccone, Gonzalo Tejeda, Andrew B. Tobin, Massimo Vassalli, Anthony J. Chalmers, Jinendra Ekanayake, Daniele Faccio

**Affiliations:** 1 Advanced Research Centre, School of Physics and Astronomy, University of Glasgow, United Kingdom; 2 Wolfson Wohl Cancer Research Centre, School of Cancer Sciences, University of Glasgow, United Kingdom; 3 Advanced Research Centre, James Watt School of Engineering, University of Glasgow, United Kingdom; 4 Institute for Bioengineering of Catalonia (IBEC), The Barcelona Institute for Science and Technology (BIST), Barcelona, Spain; 5 Advanced Research Centre, School of Molecular Biosciences, University of Glasgow, United Kingdom; 6 Stanford Neuroscience Health Center, Stanford University, USA

## Abstract

This erratum corrects formatting errors in Figs. 1, 2, and 4 of our previously published article ‘Wide-Field and Non-Invasive Imaging of Brain Tumours with Scattered Light Techniques’ [ Biomed. Opt. Express 17, 1112 (2026)10.1364/BOE.58740741970562 PMC13064597]. These errors do not affect the accuracy or significance of the results.

## 1. Correction

1. In Fig. 1(b) (top right) in [1], the brain image had shifted partially out of frame. In the bottom right panel, the colourbar contained an unintended thick blue band at the top. These errors have been corrected in the updated figure.
2. In Fig. 2, the brain images had shifted partially out of frame. The colourbars contained an unintended blue band at the top. These errors have been corrected in the updated figure.
3. In Fig. 4, the brain images had shifted partially out of frame. The colourbars contained an unintended thick blue band at the top. These errors have been corrected in the updated figure.

**Fig. 1.**
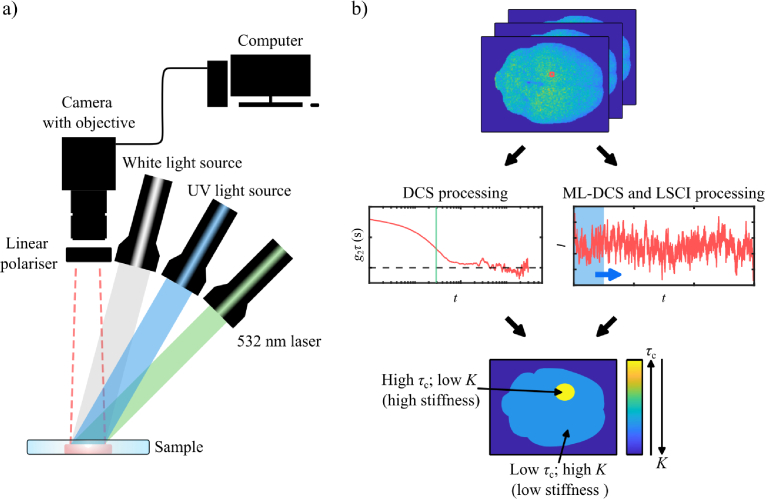
**Experimental Setup and Data Processing Pipeline.** a) A diagram of the imaging apparatus. Illumination is performed sequentially using a white light source for a white light image, a UV light source for a fluorescence image, and a laser source for the DCS, ML-DCS and LSCI images. The illumination sources were positioned at an oblique angle to the sample. A CMOS camera imaged the light reflected and scattered off the sample, and a computer performed computational imaging with this light. Light from the sample was cross-polarised (10,000:1 extinction ratio) to reduce specular reflections from the sample. Here, linearly polarised light illuminated the sample, and a linear polariser was mounted on the camera objective and turned until specular reflections were eliminated from the brain’s image before speckle acquisition. b) A speckle series of the brain obtained by laser light illumination is shown with a single pixel highlighted in red. For DCS processing, the $\tau_{c}$ of the sample pixel is found by means of $g_{2}$ time series autocorrelation. For LSCI, a window is scanned along the time series and K is found, respectively. The window size is different for both techniques. The above processes are performed for each pixel, and the output image is a colourmap of either $\tau_{c}$ or K .

**Fig. 2.**
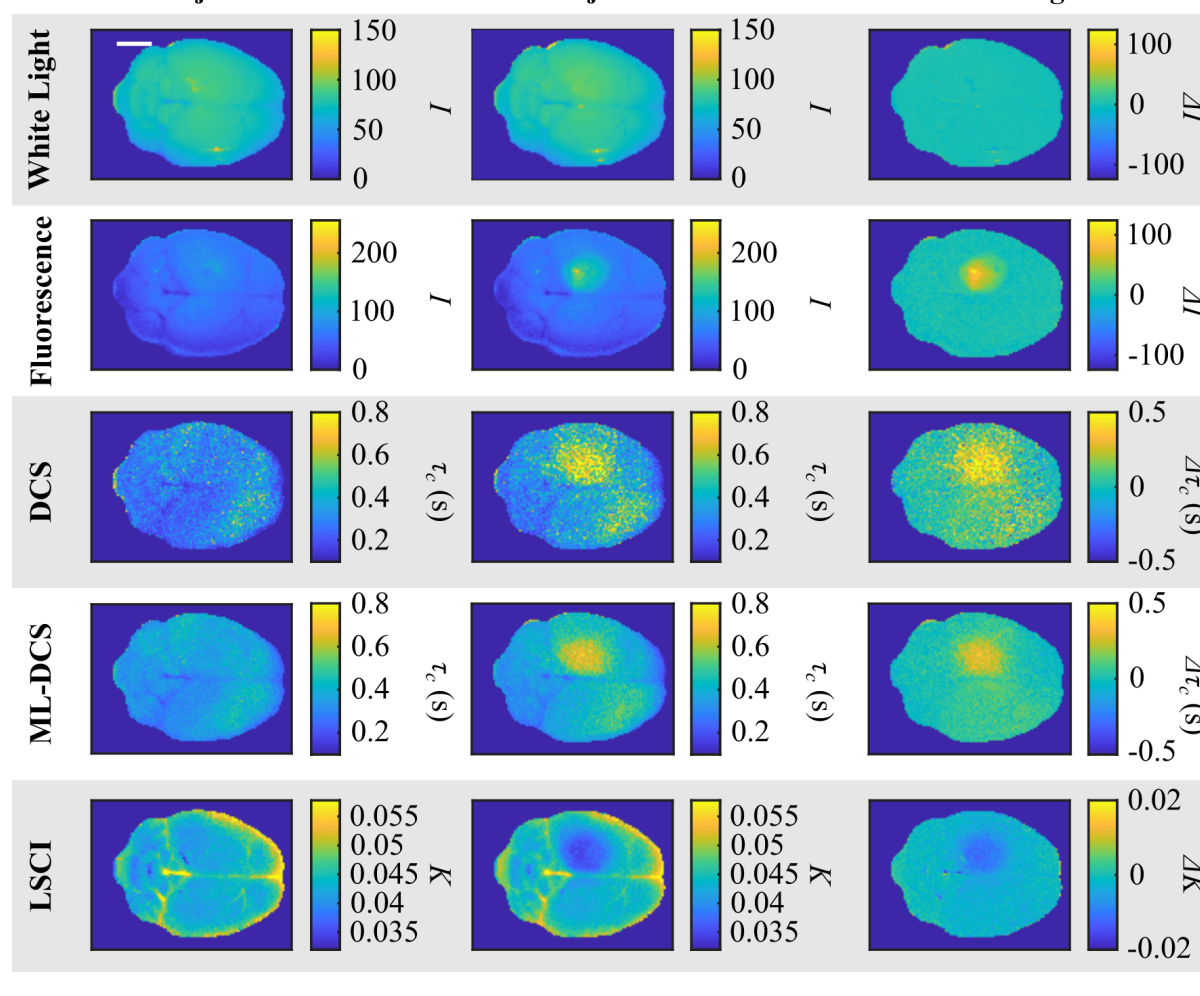
**Tumour Proxy Model DCS, ML-DCS, and LSCI Results.** Row headings indicate imaging methods of white light, fluorescence, DCS, ML-DCS, and LSCI. Column headings indicate if images were taken pre-injection and post-injection of the PFA-DAPI tumour proxy. The last column shows a difference image of the post- and pre-injection images. The scale bar on the top left panel is equivalent to 5 mm and applies to all other panels.

**Fig. 4.**
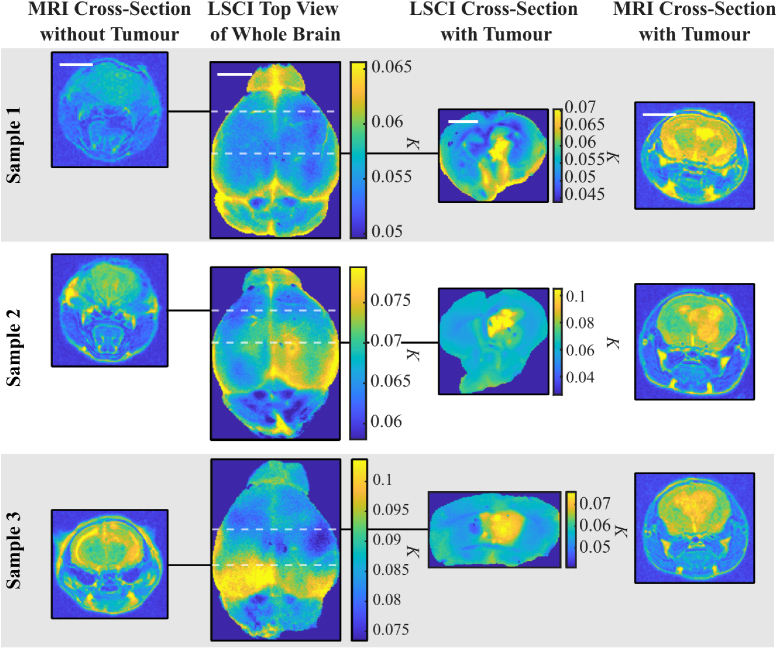
**Tumour Model LSCI and MRI Results.** Whole brain LSCI images are shown for three mouse brain samples. Corresponding cross-sectional images for LSCI and MRI are also shown, which show slices of the brain with (right hand side images) and without (left hand side images) a tumour. Tumours appear as a hotspot in the LSCI maps indicating they are softer than the healthy tissue. Sample 3 shows signs of hydrocephalus through MRI in the brain’s left hemisphere, which is also detected as a high contrast, low stiffness hot spot in the LSCI top view image. White scale bars are equivalent to 5 mm. Note that colourbar ranges are different for each panel.

## Data Availability

Data underlying the results presented in this work are available at [4].
