# Supplementary material for: Wide-field and non-invasive imaging of brain tumours with scattered light techniques
Source: Biomed Opt Express. 2026 Feb 2;17(3):1112–24. doi: 10.1364/BOE.587407 (PMC13064597; doi:10.1364/BOE.587407)
Supplement: Supplementary file 1 [file boe-17-3-1112-s001.pdf]

Supplemental Document

Biomedical Optics EXPRESS

Wide-field and non-invasive imaging of brain tumours with scattered light techniques: supplement

PHILIP BINNER,[1] JACK RADFORD,[1] ILYA STARSHYNOV,[1] MANSA MADHUSUDAN,[1] KAREN STRATHDEE,[2] KATRINA STEVENSON,[2] MATTHEW WALKER,[3] GIUSEPPE CICCONE,[3,4] GONZALO TEJEDA,[5] ANDREW B. TOBIN,[5] MASSIMO VASSALLI,[3] ANTHONY J. CHALMERS,[2] JINENDRA EKANAYAKE,[6] AND DANIELE FACCIO[1,*]

[1] *Advanced Research Centre, School of Physics and Astronomy, University of Glasgow, United Kingdom*
[2] *Wolfson Wohl Cancer Research Centre, School of Cancer Sciences, University of Glasgow, United Kingdom*
[3] *Advanced Research Centre, James Watt School of Engineering, University of Glasgow, United Kingdom*
[4] *Institute for Bioengineering of Catalonia (IBEC), The Barcelona Institute for Science and Technology (BIST), Barcelona, Spain*
[5] *Advanced Research Centre, School of Molecular Biosciences, University of Glasgow, United Kingdom*
[6] *Stanford Neuroscience Health Center, Stanford University, USA*
[*] *Daniele.Faccio@glasgow.ac.uk*

This supplement published with Optica Publishing Group on 2 February 2026 by The Authors under the terms of the Creative Commons Attribution 4.0 License in the format provided by the authors and unedited. Further distribution of this work must maintain attribution to the author(s) and the published article's title, journal citation, and DOI.

Supplement DOI: https://doi.org/10.6084/m9.figshare.31132267

Parent Article DOI: https://doi.org/10.1364/BOE.587407

Wide-Field and Non-Invasive Imaging of Brain Tumours with Scattered Light Techniques: Supplemental Document

A. The ML-DCS Model

The machine learning diffuse correlation spectroscopy (ML-DCS) model is used to reduce the acquisition time and speed up the process of diffuse correlation spectroscopy (DCS). The ML-DCS model architecture is illustrated in Fig. S1 a). It is a supervised learning model adapted from [1] that maps short intensity time series data to speckle decorrelation time values τ_c, as measured by DCS. The model consists of two convolutional layers and six dense layers, represented by blue and orange boxes, respectively. The convolutional layers apply convolutional filters to extract features from the time series data. The output features of the convolutional layers are then flattened and mapped to unique speckle decorrelation times through the dense layers. Additionally, stride lengths of 10 and dropout regularisation were also incorporated into the model. Gradient descent was used to minimise the mean absolute percentage error and train the model with the Adam optimiser. Lastly, our model uses a learning rate of 10^{-5}, a batch size of 256, and 200 training epochs per run. Training was performed until the mean absolute percentage error fell below 5% with no signs of overfitting, such as the training and validation loss curves straying from one other. The training was performed on a desktop with the following characteristics: Intel i9-10900X CPU (3.7 GHz), 256 GB RAM, and an NVIDIA GeForce RTX3090 with 24 GB of memory. The Keras and TensorFlow packages were used.

B. Generation of Synthetic Time Series Data

Synthetic data was used to train the ML-DCS model and was generated through light scattering simulations and the coupled dipole approximation (CDA) [2–5]. CDA models a bulk scattering medium composed of many dipoles that radiate an electric field when excited by an external field. One can obtain the field from a scattering medium and therefore a time varying speckle pattern if that scattering medium is also dynamic.

In simulating light scattering with CDA, one must define the external field, the position of scattering dipoles, and the interaction between all scatterer dipole pairs. In our code, we set the scatterer polarisability to a constant and designate that scatterers are only excited at their resonant frequency, $\omega = \omega_0$. The equation below describes the polarisability,

$$\alpha(\omega)\big|_{\omega\rightarrow\omega_0} \sim -\frac{2\pi\mathcal{L}}{k_0^3(\omega-\omega_0+i\mathcal{L}/2)} = -\frac{4\pi}{ik_0^3}. \quad \text{(S1)}$$

Here, $\mathcal{L}$ is the laser linewidth and K_0 is the wavenumber of the external field.

The incident light is represented by a vertically polarised plane wave moving from left to right along x, and is described by,

$$E_0(x) = \exp\left[-ik_0x\right]. \quad \text{(S2)}$$

The field at scatterer m will be a sum of the incident field and every other field generated by the other scatterers,

$$E_m = E_0(r_m) + \alpha(\omega)k_0^2 \sum_{n=1,n\neq m}^{N_s} G_0(k_0|\mathbf{r}_n-\mathbf{r}_m|)E_n. \quad \text{(S3)}$$

Here, the free-space Green's function describes the field that each scatterer m creates over all space,

$$G_0(\mathbf{r}_0,\mathbf{r}) = -\frac{\exp\left[ik_0|\mathbf{r}_0-\mathbf{r}|\right]}{4\pi|\mathbf{r}_0-\mathbf{r}|}. \quad \text{(S4)}$$

For a vector or unknown fields, $\epsilon = E_1, ..., E_n, ..., E_{N_s}$, the Eqn. S3 can be expressed in matrix notation and computationally solved for ϵ by inverting G_0,

$$-\epsilon_0 = \alpha(\omega)k_0^2(\mathcal{G}_0 - \mathbb{I})\epsilon, \quad \text{(S5)}$$

where $\mathcal{G}_{0,jk} = G_0(k_0|\mathbf{r}_j - \mathbf{r}_k|)$. Once obtaining ϵ, the field at any point in space can be found through,

$$E(\mathbf{r}) = E_0(\mathbf{r}) + \alpha(\omega)k_0^2 \sum_{k=1}^{N_s} G_0(k_0|\mathbf{r} - \mathbf{r}_k|)E_k. \quad \text{(S6)}$$

The speckle intensity can then be found by $|E(\mathbf{r})|^2$.

Lastly, the scattering medium can be made dynamic by changing the coordinates of the scatterers iteratively. For example, the scattering medium can undergo Brownian motion, as described by the following equation,

$$\mathbf{r}_{i,j,k} = \mathbf{r}_{i-1,j-1,k-1} + \sqrt{2D\Delta t}w_{i,j,k}. \quad \text{(S7)}$$

Here, D is a diffusion constant, Δt is a time step smaller than the characteristic timescale of the Brownian motion, and $w_{i,j,k}$ is a pseudorandom number [6].

$64 \times 64 \times 200,000$ (x pixels, y pixels, frames) speckle time series were created through CDA simulations. The g_2 autocorrelation function was applied the output speckle time series to obtain a τ_c for each time series. The distribution of τ_c values is illustrated in Fig. S1 b) and represents a Gaussian distribution. To remove bias in the ML-DCS model, this distribution should be uniform across a range of τ_c. It would take a considerably long time to achieve a uniform τ_c distribution through multiple CDA simulations, and we found that an efficient way to simulate a broader range of τ_c values was to upsample time series by a factor, u, which also effectively increased the corresponding τ_c of that time series by u. Upsampling was performed through MATLAB's resample function. A more uniform distribution of τ_c is shown in Fig. S1 c), which is cropped between 0.2 and 2 s, covering most τ_c that we have measured pertaining to an ex vivo brain. Fig. S1 d) shows a linear dependence between τ_c and the upsampling factor u, as found through upsampling and applying the g_2 autocorrelation to a single time series. This shows that it is practical to upsample time series to create slower decorrelation time series. Lastly, Fig. S1 e) shows three examples of different τ_c and their corresponding u and time series. These τ_c and time series are then used to train the ML-DCS model in Fig. S1 a). Application of CDA and training of the ML-DCS model is described in more detail in [7].

C. Predictions of the ML-DCS Model on PFA-Injected Brains

Once the ML-DCS model is trained, it can be applied to real speckle time series data, so long as the decorrelation time of the sample in question sits in the pre-defined τ_c range of the model. Here, our model was trained for $0.2 < \tau_c < 2$ s and previous experiments with mouse brain have resulted in $\tau_c \sim 1$ s. For noisy real data, one can apply a Butterworth filter to smooth the time series and make it more similar to synthetic training data. We use Scipy's lowpass Butterworth filter of order 1 and a critical frequency 0.1 for our data.

Results of the ML-DCS model are shown in Fig. S2. Firstly, on the left is a single frame ML-DCS output image of a paraformaldehyde (PFA)-injected mouse brain, equivalent to 10 s of brain speckle acquisition. On the right is an average output image that represents the average of 5000 model predictions and is equivalent to 60 seconds of acquisition. Note that this image is also shown in the main document. We conclude that one is able to identify the stiffened tumour region with a short 10 s acquisition; however, better signal-to-noise ratio (SNR) is obtained by averaging multiple output frames of the ML-DCS model.

D. Predictions of the ML-DCS Model on Mouse Tumour Model

Fig. S3 shows the top view and cross-sectional images obtained by the ML-DCS model for the three mouse tumour model samples that are reported in the main document. In sample 1, the presence of the tumour using the ML-DCS cannot be discerned in the top view image. Furthermore, in the cross-sectional image, the tumour is again difficult to make out, suggesting its stiffness is similar to the surrounding healthy tissue. However, in samples 2 and 3, the contrast in tumour and healthy tissue is stronger. For sample 2's top view image, we observe lower τ_c in both hemispheres of the brain, indicative of softer tissue and similar to the laser speckle contrast imaging (LSCI) results in the main document. The ML-DCS cross-section tumour region consists of areas of high τ_c surrounded by lower τ_c suggesting that the tumour consists of both stiff and soft regions, perhaps owing to necrotic and living tumour tissue. However, the current results and mechanisms that drive tumour growth and decay are inconclusive. Lastly, the ML-DCS top view and cross-sectional images for sample 3 agree well with the LSCI results in the main document. Soft tissue appears as low τ_c for the ML-DCS method, while high K is seen for the

LSCI method. In summary, the ML-DCS method is able to locate a tumour based on relative stiffness differences between the tumour and healthy tissue.

REFERENCES

1. M. G. Smith, J. Radford, E. Febrianto, *et al.*, "Machine learning opens a doorway for microrheology with optical tweezers in living systems," AIP Adv. **13**, 075315 (2023).
2. M. Lax, "Multiple scattering of waves," Rev. Mod. Phys. **23**, 287–310 (1951).
3. M. Lax, "Multiple scattering of waves. ii. the effective field in dense systems," Phys. Rev. **85**, 621–629 (1952).
4. N. Fayard, A. Cazé, R. Pierrat, and R. Carminati, "Intensity correlations between reflected and transmitted speckle patterns," Phys. Rev. A **92** (2015).
5. I. Starshynov, "Quantum and classical correlations of multiply scattered light," Thesis, University of Exeter (2018).
6. G. Volpe and G. Volpe, "Simulation of a brownian particle in an optical trap," Am. J. Phys. **81**, 224–230 (2013).
7. P. Binner, "Novel applications and improvements to the diffuse correlation spectroscopy technique," Ph.D. thesis, University of Glasgow (2024).

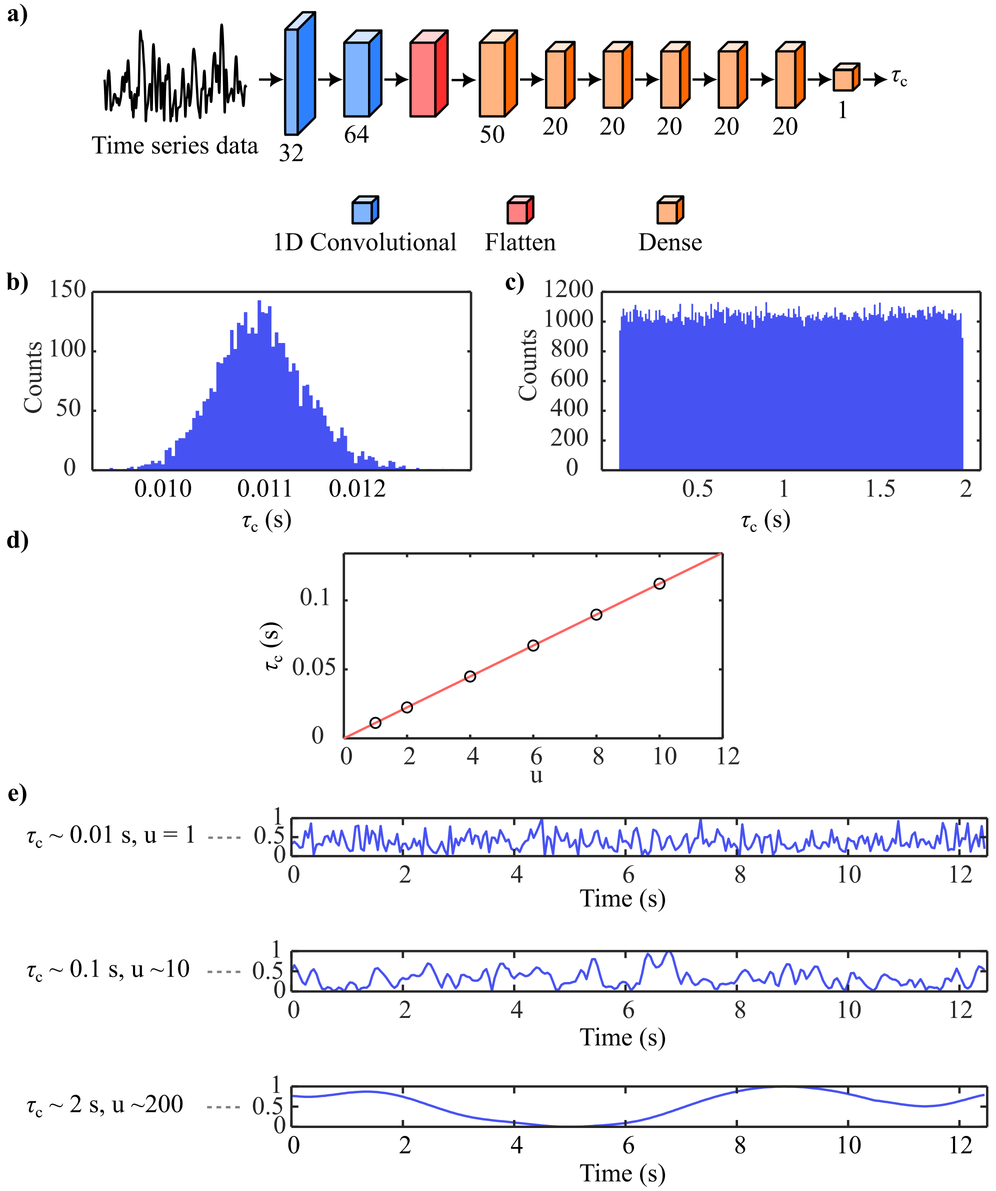

Fig. S1. Simulation of synthetic data and ML-DCS model architecture. a) The ML-DCS model architecture. Two convolutional layers are shown in blue (32 kernels × 10 frames for first layer and 64 kernels × 100 frames for second layer). b) A distribution of τ_c for speckle time series made by CDA simulations. c) A uniform distribution of τ_c from upsampled speckle time series and cropped between 0.2 and 2 s. d) τ_c vs upsampling factor u showing a linear dependence. e) Three simulated intensity time series examples with corresponding τ_c and u. Figure adapted from [7].

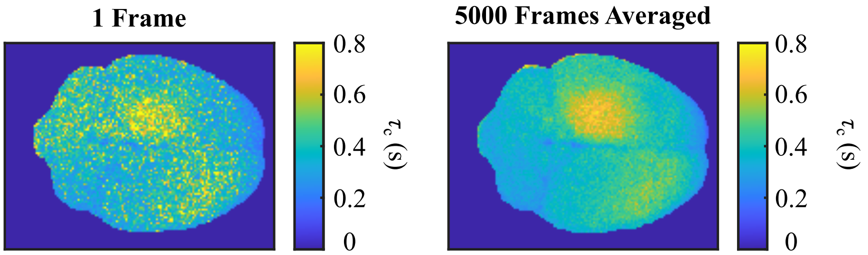

Fig. S2. Predictions of the ML-DCS Model on PFA-Injected Mouse Brain. On the left is a single frame prediction of the ML-DCS model, and on the right is an average prediction from 5000 frames.

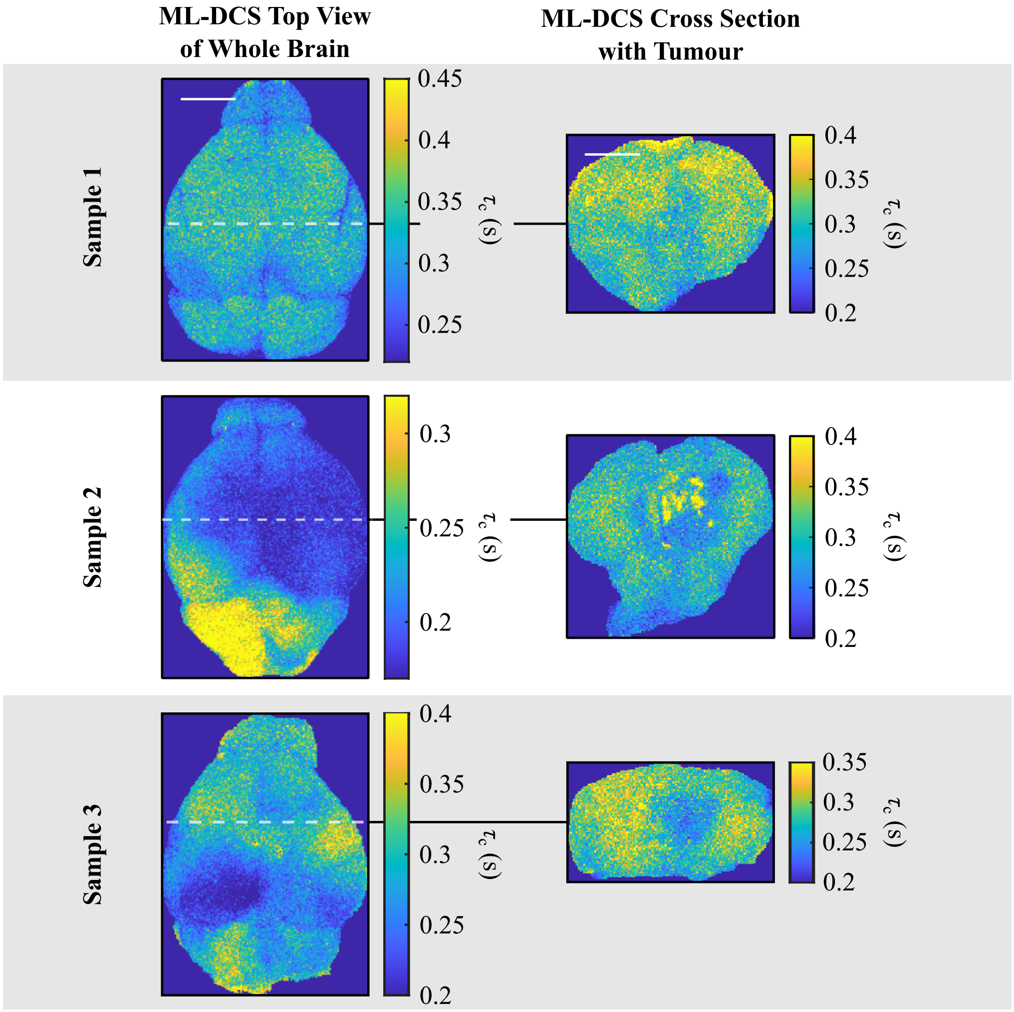

Fig. S3. Predictions of the ML-DCS Model on Mouse Tumour Model. Top view images of mouse brains constructed by averaging 5000 output frames of ML-DCS model are shown on the left. On the right are cross-sections whose axial positions are shown by the horizontal white dashed lines in the respective top view images. Scale bars on the top most images represent 5 mm.
